# Associations between Intensity, Frequency, Duration, and Volume of Physical Activity and the Risk of Stroke in Middle- and Older-Aged Chinese People: A Cross-Sectional Study

**DOI:** 10.3390/ijerph17228628

**Published:** 2020-11-20

**Authors:** Donghui Yang, Yuqian Bian, Zixin Zeng, Yiran Cui, Yafeng Wang, Chuanhua Yu

**Affiliations:** 1 Department of Epidemiology and Biostatistics, School of Health Sciences, Wuhan University, Wuhan 430071, China; yangdh@whu.edu.cn (D.Y.); zzx7021@whu.edu.cn (Z.Z.); 2019283050055@whu.edu.cn (Y.C.); wonyhfon@whu.edu.cn (Y.W.); 2 Information Management and Information System, School of Medical and Health Management, Huazhong University of Science and Technology, Wuhan 430030, China; byq847660458@163.com

**Keywords:** association, physical activity (PA), stroke risk, middle- and older-aged Chinese

## Abstract

Context: Persuasive evidence has shown the inverse associations between physical activity (PA) and the risk of stroke. However, few studies have investigated the associations between different dimensions (intensity, frequency, duration, volume) of PA and the risk of stroke. Objective: To investigate the associations between different dimensions of PA and the risk of stroke in total participants and subgroups. Method: This study included 6250 individuals aged 45 years old and above from the China Health and Retirement Longitudinal Study (CHARLS). PA was divided into vigorous PA (VPA), moderate PA (MPA), and light PA (LPA), and described in different dimensions (intensity, frequency, duration, volume). Stroke was defined on the basis of self-reported diagnosis and related treatments. Binary logistic regression models were established to assess the associations between different dimensions of PA and the risk of stroke in total participants and subgroups stratified by sex. Results: Individuals taking VPA with a frequency of 3–5 d/w, duration of ≥240 min/d, volume of ≥300 min/w had lower risks of strokes in total participants (Odds ratio (OR) = 0.32, 95% confidence interval (CI): 0.13, 0.75; OR = 0.60, 95% CI: 0.38, 0.94; OR = 0.68, 95% CI: 0.46, 0.99, respectively). However, significant associations of VPA with the risk of stroke in men were only observed in the duration of ≥240 min/d and volume of ≥300 min/w (OR = 0.53, 95% CI: 0.30, 0.93; OR = 0.61, 95% CI: 0.38, 0.99, respectively) whereas no significance in women. Compared with individuals taking no MPA, inverse significant associations between the risk of stroke and any level of frequency, duration and volume in MPA were observed in total sample (OR ranging from 0.16–0.40, all *p* < 0.05), whereas significant associations between the risk of stroke and MPA were found in men except the duration of 10–29 min/d and volume of 150–299 min/w (OR ranging from 0.26–0.35, all *p* < 0.05), and in women except the frequency of 1–2 d/w and duration of ≥240 min/d (OR ranging from 0.14–0.49, all *p* < 0.05). No significant associations could be observed in total participants and subgroups between LPA and the risk of stroke. Conclusion: This study revealed some significant associations between different dimensions of PA, especially MPA, and the risk of stroke. Furthermore, the difference of association was observed in the groups with different sex. Further prospective study is needed to determine deeper associations between PA and the risk of stroke.

## 1. Introduction

Stroke was one of the leading causes of death, accounting for 10% of all deaths worldwide in 2016 [1]. Additionally, the age-standardized stroke prevalence rate increased by 3.1% from 1990 to 2017, up to 1300.6 per 100,000 in 2017 [2]. Globally, from 1990 to 2016, the estimated lifetime risk of stroke increased from 22.8% to 24.9%, a relative increase of 8.9%, among the group aged 25 years old and above [3]. In China, stroke ranked as the third-highest cause of death, resulting in 1.57 million deaths in 2018 [4]. In addition, the prevalence of stroke has increased over the previous 30 years up to 1596.0 per 100,000 people in 2013 [5]. It was estimated that China had the highest estimated lifetime risk of stroke among the group aged 25 years old and above, up to 39.3% [3].

Many factors are related to the risk of stroke such as hypertension, diabetes, physical inactivity, etc., and around 80% of strokes were attributable to these modifiable risk factors [6]. Physical activity (PA) was defined as any bodily movement produced by skeletal muscles that result in energy expenditure [7]. Some studies demonstrated mechanisms that PA could reduce the risk of cardiovascular events [8,9]. Up to now, many studies have found an inverse association of PA with the risk of stroke [10,11,12]. A study showed that a high or moderate level of PA was protective against stroke in men not women [13]. In addition, a study found that vigorous physical activity (VPA) was associated with a lower chance of having a stroke compared with light physical activity (LPA) [14], whereas other research has shown that VPA did not decrease the risk of stroke [15]. Meanwhile, there were also some inconsistent results in several studies about the associations between the frequency of PA and the risk of stroke [16,17]. A study found that PA frequency was not associated with the risk of coronary heart disease (CHD) [16], whereas another study showed even a low frequency of PA could prevent stroke [17]. Although many studies have estimated the association of PA with the risk of stroke, associations between comprehensive dimensions of PA and the risk of stroke are rarely investigated.

Therefore, the aim of this study was to estimate associations of the risk of stroke with different dimensions of PA and compare whether there are differences in association of the stroke and PA between men and women.

## 2. Materials and Methods

### 2.1. Study Population

In this study, we used the data from the China Health and Retirement Longitudinal Study (CHARLS) conducted in 2015. CHARLS is a nationally representative longitudinal cohort study of individuals aged 45 years old and above in China. Its baseline survey was conducted in 2011–2012 and further follow-up survey was conducted every two years. In order to estimate the social, economic, and health status, and to establish a high qualitative database, CHARLS selected participants in 450 communities of 150 county-level units from 28 provinces chosen in China based on a four-stage stratified cluster sampling method. The ethics committee of Peking University Health Science Center has approved the CHARLS. The ethical approval number was IRB00001052-11015. More detailed contents of CHARLS have been previously documented [18].

Among 20,967 participants in 2015, we excluded 1277 participants under 45 years old or missing age data, 2855 participants missing a diagnosis of stroke, and 9132 individuals missing information on frequency and/or duration of VPA/moderate physical activity (MPA)/LPA. Meanwhile, we removed 1453 participants lacking data on sex, educational status, marital status, drinking, smoking, Body Mass Index (BMI), case weights, and abnormal values of BMI (>100 kg/m^2^). Finally, 6250 participants were included in this cross-sectional study.

### 2.2. Stroke Measurements

Stroke was defined through the question “Have you been diagnosed with a stroke by a doctor?” and “Are you now taking any of the following treatments because of your stroke?”. We considered that the individual had had a stroke when the response was “yes” to either or both of the questions.

### 2.3. Assessment of PA

Each subject was asked, “Do you conduct PA for at least 10 min continuously in a usual week?” for each category: (1) Doing VPA that makes breathing much harder than normal; (2) Doing MPA that makes breathing somewhat harder than normal; (3) Doing LPA such as walking. If the response was “no”, they would be considered as taking no VPA/MPA/LPA. If the answer was “yes”, they would be further inquired “How many days do you normally take VPA/MPA/LPA in a week?” and “How long do you spend on VPA/MPA/LPA each time?”.

Frequency of PA ranged from 0–7 d/w in all three intensities of PA and was separated into 4 levels: no activity (0 d/w); 1–2 d/w; 3–5 d/w; and 6–7 d/w. Duration of PA was categorized into 5 levels in accordance with CHARLS: no activity; 10–29 min/d; 30–119 min/d; 120–239 min/d; and ≥240 min/d, considering some participants taking no PA or spending too long each time. It was suggested that if the elderly do VPA at least 75 min/w, or MPA at least 150 min/w and VPA/MPA over 300 min/w, this may cause harm to the senior individuals [7]. Therefore, we calculated the total volume of VPA/MPA/LPA in a week by multiplying frequency and duration of VPA/MPA/LPA. In this study, referring to the WHO guideline [7], volume of VPA was divided into 4 levels: no activity; 10–74 min/w; 75–299 min/w; and ≥300 min/w, whereas volume of MPA was separated into 4 levels; no activity; 10–149 min/w; 150–299 min/w; and ≥300 min/w. Despite no guidelines on LPA, we divided volume of LPA into 4 levels like MPA: no activity; 10–149 min/w; 150–299 min/w; and ≥300 min/w.

### 2.4. Assessment of Covariables

Information of individuals on social-demographic factors, health behaviors and health related status was collected via questionnaire-based interviews in 2015. Social-demographic factors included age (continuous variable), sex (male, female), educational status (junior high school or below; senior high school or vocational school; college or above), marital status (married or partnered; separated, divorced or widowed; never married). Health behaviors included: drinking (never; former; current), smoking (never; former; current). Health related status included BMI (continuous variable).

### 2.5. Data Analysis

Categorical variables were expressed as frequencies and percentages. Continuous variables were described as a mean value ± standard deviation (SD). Associations of different dimensions of PA with the risk of stroke were estimated by binary logistic regression. Odds Ratios (ORs) and 95% confidence intervals (CIs) were calculated to measure the effects of different dimensions of PA. Model 1 presented the key potential confounding covariables—age, sex, educational status, marital status, drinking, smoking, BMI—and case weights adjusted results in total participants. Case weights were used to adjust for the stratified sampling method and patterns caused by non-response individuals in this study. Model 2 and model 3 presented key covariables and case weight-adjusted results in men and women, respectively. In order to reduce the selection bias, we excluded 502 participants with PA disability and established model 4 to reduce the influence of movement restrictions. Furthermore, to evaluate the robustness of the results in this study, we calculated the *E*-value [19]. Assessed E -value indicated the minimum strength of association between unmeasured confounders, PA, and the risk of stroke. Data were analyzed with Stata IC 15 (StataCorp, College Station, TX, USA). If *p*-value < 0.05, it was considered statistically significant in all analyses.

## 3. Results

### 3.1. Characteristics of the Participants

Our study included 6250 participants, which consisted of 228 stroke patients and 6022 individuals without a stroke. Compared with individuals without a stroke, stroke patients were older and a higher percentage were men. Participants with a stroke diagnosis had a higher BMI and a greater proportion were separated, divorced, widowed, or had never married, and were junior high school or below in educational status. Additionally, the proportion of former alcohol consumption, and former and current smoker was higher in participants with stroke than those without stroke. The basic characteristics of the participants are shown in Table 1.

### 3.2. Frequency of PA and the Risk of Stroke

**Figure 1:**
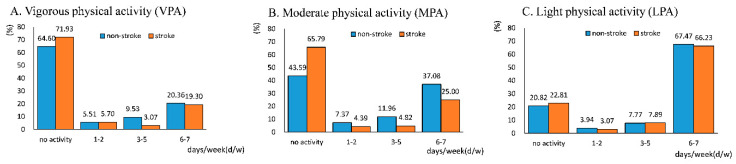
Frequency of vigorous physical activity (VPA) (**A**), moderate physical activity (MPA) (**B**), and light physical activity (LPA) (**C**) in participants with and without stroke. The height of the column represents the proportion of individuals at this level of frequency in total participants with and without a stroke, respectively. Corresponding proportion is shown above the column.

Regarding the frequency of PA, the majority of individuals took no PA or frequent PA (6–7 d/w) in all three intensities of PA. With the intensity decreasing, the proportion of active individuals increased in participants both with and without stroke (35.40% for VPA, 56.41% for MPA, 79.18% for LPA in participants with stroke; 28.07% for VPA, 34.21% for MPA, 77.19% for LPA in participants without stroke). Participants with a stroke were more inactive in VPA and MPA, whereas individuals at different levels of frequency in LPA occupied a similar proportion in total participants with and without a stroke. Frequency of PA in participants with and without stroke a is shown in Figure 1.

Compared with inactive participants, individuals taking VPA 3–5 d/w had a lower chance of suffering from a stroke (Odds ratio (OR) = 0.32, 95% confidence interval (CI): 0.13, 0.75), whereas a significant association was not observed in the subgroups. Concerning MPA, lower risks of stroke were associated with MPA in all levels of frequency in the whole sample (OR = 0.35, 95% CI: 0.18, 0.72 for 1–2 d/w, OR = 0.23, 95% CI: 0.12, 0.46 for 3–5 d/w, OR = 0.40, 95% CI: 0.27, 0.58 for 6–7 d/w) and men (OR = 0.26, 95% CI: 0.09, 0.78 for 1–2 d/w, OR = 0.28, 95% CI: 0.12, 0.63 for 3–5 d/w, OR = 0.34, 95% CI: 0.20, 0.60 for 6–7 d/w). However, the inverse significant associations were only observed in the women taking MPA with a frequency of 3–5 d/w and 6–7 d/w (OR = 0.14, 95% CI: 0.04, 0.46 for 3–5 d/w, OR = 0.46, 95% CI: 0.28, 0.78 for 6–7 d/w). There was no statistically significant association between the frequency of LPA and the risk of stroke in total participants and subgroups. Associations between the frequency of PA and the risk of stroke are shown in Table 2.

### 3.3. Duration of PA and the Risk of Stroke

**Figure 2:**
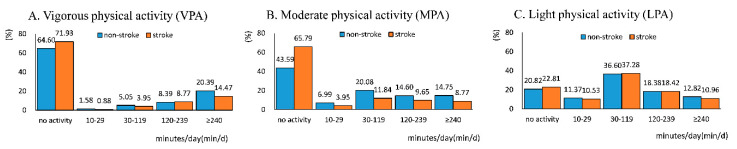
Duration of VPA (**A**), MPA (**B**), and LPA (**C**) in participants with and without a stroke. The height of the column represents the proportion of individuals at this level of duration in total participants with and without a stroke, respectively. Corresponding proportion is shown above the column.

Regarding the duration of PA, the majority of active participants took VPA with a duration of ≥240 min/d, and MPA and LPA with a duration of 30–119 min/d. With the length of duration increasing, the proportion of individuals taking PA increased in VPA whereas it decreased in MPA and LPA, except for the duration of 10–29 min/d. The duration of PA in participants with and without stroke is shown in Figure 2.

Spending ≥240 min/w of VPA resulted in a lower risk of stroke in total participants (OR = 0.60, 95% CI: 0.38, 0.94) and men (OR = 0.53, 95% CI: 0.30, 0.93) whereas no significance was observed in women. Meanwhile, total individuals had significantly lower odds of suffering from a stroke when taking any level of MPA (OR = 0.31, 95% CI: 0.14, 0.65 for 10–29 min/d, OR = 0.32, 95% CI: 0.20, 0.53 for 30–119 min/d, OR = 0.40, 95% CI: 0.24, 0.67 for 120–239 min/d, OR = 0.40, 95% CI: 0.23, 0.68 for ≥240 min/d). In the analysis stratified by sex, significant associations were observed in all MPA categories except 10–29 min/d in the group of men (OR = 0. 27, 95% CI: 0.13, 0.56 for 30–119 min/d, OR = 0.35, 95% CI: 0.17, 0.72 for 120–239 min/d, OR = 0.34, 95% CI: 0.17, 0.68 for ≥240 min/d), whereas significant associations were observed in all MPA categories except ≥240 min/d in the group of women (OR = 0.27, 95% CI: 0.10, 0.75 for 10–29 min/d, OR = 0.38, 95% CI: 0.19, 0.75 for 30–119 min/d, OR = 0.49, 95% CI: 0.25, 0.99 for 120–239 min/d). No significant association of duration of LPA with the risk of stroke was observed in total participants and subgroups. Associations between duration of PA and the risk of stroke are shown in Table 2.

### 3.4. Volume of PA and the Risk of Stroke

**Figure 3:**
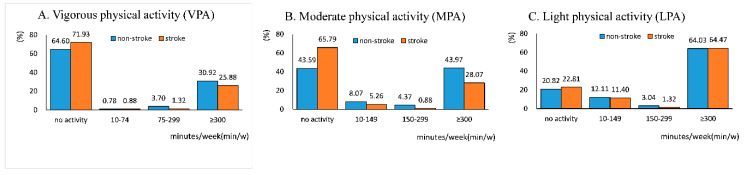
Volume of VPA (**A**), MPA (**B**), LPA (**C**) in participants with and without a stroke. The height of the column represents the proportion of individuals at this level of volume in participants with and without a stroke, respectively. Corresponding proportion is shown above the column.

Regarding the volume of PA, stroke patients took less VPA/MPA, whereas the proportion distribution of LPA volume was similar in the group with and without a stroke. Of the individuals without a stroke, 34.62% had reached the threshold of 75 min/w of VPA and 48.34% had reached the threshold of 150 min/w of MPA, whereas 27.1% reached the threshold of 75 min/w of VPA and 28.95% had reached the threshold of 150 min/w of MPA among stroke patients. The volume of PA in participants with and without stroke is shown in Figure 3.

Taking VPA with the volume of ≥300 min/w was associated with lower odds of having a stroke in total participants (OR = 0.68, 95% CI: 0.46, 0.99) and men (OR = 0.61, 95% CI: 0.38, 0.99). Among the participants taking MPA, any level of volume of MPA was associated with a lower risk of stroke for all participants (OR = 0.34, 95% CI: 0.18, 0.66 for 10–149 min/w, OR = 0.16, 95% CI: 0.04, 0.69 for 150–299 min/w, OR = 0.38, 95% CI: 0.26, 0.55 for ≥300 min/w). Performing MPA for 10–149 min/w and ≥300 min/w resulted in both men and women having a lower risk of stroke (men: OR = 0.34, 95%: 0.12, 0.92 for 10–149 min/w, OR = 0.32, 95%: 0.19, 0.54 for ≥300 min/w; women: OR = 0.35, 95% CI: 0.15, 0.84 for 10–149 min/w, OR = 0.47, 95% CI: 0.29, 0.78 for ≥ 300 min/w). There was no significant association between the volume of LPA and the risk of stroke in total participants and subgroups. The associations between the volume of PA and the risk of stroke are shown in Table 2.

### 3.5. Sensitivity Analysis

Excluding those with PA disability, we extracted 5748 participants from 6250 individuals. Compared with the results in participants without exclusions, significant associations were not observed in the duration of ≥240 min/d and the volume of ≥300 min/w of VPA. Appendix A Table A1 presented results after excluding respondents with disability. Robustness to the association was estimated using the *E*-value. The observed significant ORs of 0.32, 0.35, 0.23, 0.40, 0.60, 0.31, 0.32, 0.40, 0.40, 0.68, 0.34, 0.16 and 0.38 could be explained by unmeasured confounders, respectively. Moreover, different dimensions of PA and the risk of stroke had an OR of at least 5.70, 5.16, 8.16, 4.44, 2.72, 5.91, 5.70, 4.4, 4.44, 2.30, 5.33, 11.98 and 4.70, respectively, beyond the measured confounders. The CI of the *E*-value was calculated to obtain the null by an unmeasured confounder associated with both PA and the risk of stroke by OR of at least 2.00, 2.12, 3.77, 2.84, 1.32, 2.45, 3.18, 2.35, 2.30, 1.11, 2.40, 2.26 and 3.04. The results of the *E*-values are shown in Appendix A Table A2.

## 4. Discussion

Although participants with a stroke were more inactive in terms of VPA and MPA, participants with and without a stroke had similar proportions in different levels of LPA. Inverse associations between PA and the risk of stroke were observed for a moderate frequency, high duration and volume of VPA in total participants and men, not women. Nearly any level of frequency, duration, and volume of MPA was associated with a lower risk of stroke, whereas no significant association was observed in those only taking LPA.

The prevalence of individuals 45 years old and above in this study was much higher than that in 2013 (1596.0 per 100,000 in China) [5]. Meanwhile, previous studies have demonstrated that stroke patients performed less VPA and MPA [20] and levels of PA were low in stroke patients [21], which may be partly explained by the shortening of fascicles [22], accumulation of connective tissue [23], and worse passive joint and muscle stiffness [22,24] caused by strokes.

PA was associated with the risk of stroke, and difference of association could be observed in subgroups stratified by sex. Compared with those taking no VPA, participants with a frequency of 3–5 d/w, duration of ≥240 min/d, volume of ≥300 min/w had a lower chance of suffering from a stroke. However, significant associations of VPA with the risk of stroke in men were only observed for the duration of ≥240 min/d and volume of ≥300 min/w, whereas we found no statistical significance in women. These results were consistent with findings in previous studies; VPA was associated with a lower risk of strokes in men not women [14,25,26]. A possible explanation for these results is that men were more active in VPA whereas women were more active in MPA in this study. In addition, a volume of ≥300 min/w, exceeding the recommendation of doing VPA for 75–150 min/w, produced benefits for stroke prevention. However, there was no evidence available to identify whether volume of PA greater than 300 min/w produced more benefits [7]. For one thing, the WHO recommended value of movement was customized to healthy individuals, while most of the elderly in China were suffering from chronic disease [27]. Moreover, stroke has many risk factors, such as hypertension and diabetes mellitus [6]. The prevalence of hypertension and diabetes mellitus are quite high in China [28,29]. Previous studies found that PA was associated with lower risks of hypertension and diabetes mellitus [30,31,32]. Furthermore, older groups and men had higher population-attributable risks of most risk factors of strokes [33]. Consequently, more PA was needed for men to offset the harmful effects of other risk factors of strokes, such as hypertension and diabetes.

With regard to MPA, taking any level of MPA could lower the chance of having a stroke in total participants and some previous studies have also found similar results [34,35]. However, there was no clear association between the risk of stroke and MPA for a duration of 10–29 min and volume of 150–299 min/w in men, and MPA a the frequency of 1–2 d/w and duration of ≥240 min/d in women. Duration of 10–29 min/d was not associated with a lower chance of having a stroke, which may be partly explained by the fact that a small number of men exercised for a duration of 10–29 min/d, and did not reach the recommendation of MPA for at least 30 min/d. Compared with men, given that women were less likely to have a stroke overall [36], women who exercised for a duration of 10–29 min/d still had a lower risk of strokes. Women spent much more time on housework than men [37]. Although housework activities may produce health benefits, women sleeping for a short or long duration have a higher possibility of poor health [38]. Additionally, women have many sleep problems in China [39,40]. Therefore, the association between MPA with a duration of ≥240 min/d and the risk of stroke in women was not found.

With regard to LPA, compared with those taking no LPA, no significant association could be observed in any level of frequency, duration, volume of LPA in total participants and subgroups. Several previous studies have supported our results [12,41,42]. In contrast, some results in previous studies found that LPA could produce health benefits [43]. For one thing, our study did not further divide physical into occupational and leisure-time PA. For another, we do not further divide LPA into a more detailed intensity of PA. Some results found that high LPA, not low LPA was discovered to have a significant association with health [44].

Regarding the strengths in this study, as far as the authors are concerned, this study may be the first study that explores the association between comprehensive dimensions of PA and the risk of stroke. In addition, this study can reduce the influence of confounders after adjustment for many covariables—age, sex, educational status, marital status, drinking, smoking, BMI. Furthermore, we explored the association not only in total participants but also in subgroups with different sexes to explore whether a difference of association could be observed in subgroups with a different sex. Moreover, this study used a large representative sample from China, so these results can be generalized to the Chinese population. Finally, a sensitivity analysis was used in this study to reduce the selection bias and express the robustness of results, which can rarely be observed in similar studies. In this study, we found some associations between different dimensions of PA and the risk of strokes. Nevertheless, cross-sectional natures of the data limited the capability to determine the direction of association or causal pathways. Future studies that use a longitudinal cohort would be utilized to estimated causality. Meanwhile, we used self-reported data about PA which may cause recall bias and produce deviation in the results, therefore studies using accelerometry to examine PA for objective measurement are needed for more accuracy in the future. Furthermore, we only compared the difference of association between PA and the risk of stroke in men and women in this study, therefore more comparisons on the association of PA with the risk of stroke could be conducted between subgroups with different age or areas.

## 5. Conclusions

The findings of this study revealed associations between different dimensions of PA and the risk of strokes. After adjustment for potential confounders, a high duration and volume of VPA was associated with a lower risk of strokes in the total sample and men, whereas not in women. Compared with individuals taking no MPA, nearly all dimensions of MPA could be associated with a lower risk of strokes, except the duration of 10–29 min/d and volume of 150–299 min/w in men and a frequency of 1–2 d/w and duration of ≥240 min/d in women. No significant association could be found in all dimensions of LPA in the total sample and subgroups. These findings can supply more evidence on the associations between PA and the risk of strokes, and a prospective design is needed to investigate the association further deeply.

## Figures and Tables

**Table 1:** Basic characteristics of participants.

Variables	Total (*n* = 6250)	Non-Stroke (*n* = 6022)	Stroke (*n* = 228)
Age, year (mean ± SD)	61.0 ± 9.2	60.9 ± 9.2	63.9 ± 9.4
BMI, kg/m^2^ (mean ± SD)	23.9 ± 3.9	23.9 ± 3.9	24.1 ± 3.8
Sex (%)			
Male	47.0	46.6	57.9
Female	53.0	53.4	42.1
Educational status (%)			
Junior high school or below	88.9	88.9	91.2
Senior high school or vocational school	10.6	10.7	7.9
College or above	0.5	0.5	0.9
Marital status (%)			
Married or partnered	87.5	87.7	80.7
Separated, divorced, or widowed	12.1	11.8	18.4
Never married	0.5	0.4	0.9
Drinking (%)			
Never	54.5	54.7	50.9
Former	11.0	10.7	20.6
Current	34.5	34.7	28.5
Smoking (%)			
Never	56.1	56.6	45.2
Former	15.9	15.5	26.3
Current	28.0	28.0	28.5

Abbreviation: SD, standard deviation; BMI, Body Mass Index.

**Table 2:** Associations between PA and the risk of stroke.

Variables	Model 1 (*n* = 6250)			Model 2 (*n* = 2936)			Model 3 (*n* = 3314)		
	OR	95% CI		OR	95% CI		OR	95% CI	
Frequency									
VPA									
No activity	1.00			1.00			1.00		
1–2 d/w	0.92	0.46	1.81	0.68	0.29	1.6	1.56	0.54	4.54
3–5 d/w	0.32 *	0.14	0.75	0.43	0.18	1.05	N/A		
6–7 d/w	0.74	0.49	1.11	0.61	0.36	1.02	1.06	0.59	1.90
MPA									
No activity	1.00			1.00			1.00		
1–2 d/w	0.35 *	0.18	0.72	0.26 *	0.09	0.78	0.53	0.21	1.32
3–5 d/w	0.23 *	0.12	0.46	0.28 *	0.12	0.63	0.14 *	0.04	0.46
6–7 d/w	0.40 *	0.27	0.58	0.34 *	0.20	0.60	0.46 *	0.28	0.78
LPA									
No activity	1.00			1.00			1.00		
1–2 d/w	0.74	0.29	1.90	0.49	0.10	2.45	1.30	0.44	3.81
3–5 d/w	0.89	0.49	1.60	0.89	0.43	1.84	0.76	0.27	2.18
6–7 d/w	0.92	0.61	1.39	0.79	0.45	1.38	1.13	0.63	2.03
Duration									
VPA									
No activity	1.00			1.00			1.00		
10–29 min/d	0.60	0.14	2.55	0.82	0.18	3.64	N/A		
30–119 min/d	0.60	0.26	1.37	0.46	0.18	1.21	1.06	0.26	4.26
120–239 min/d	0.82	0.46	1.45	0.71	0.34	1.51	1.05	0.46	2.4
≥240 min/d	0.60 *	0.38	0.94	0.53 *	0.30	0.93	0.80	0.40	1.57
MPA									
No activity	1.00			1.00			1.00		
10–29 min/d	0.31 *	0.14	0.65	0.35	0.12	1.03	0.27 *	0.10	0.75
30–119 min/d	0.32 *	0.20	0.53	0.27 *	0.13	0.56	0.38 *	0.19	0.75
120–239 min/d	0.40 *	0.24	0.67	0.35 *	0.17	0.72	0.49 *	0.25	0.99
≥240 min/d	0.40 *	0.23	0.68	0.34 *	0.17	0.68	0.51	0.23	1.15
LPA									
No activity	1.00			1.00			1.00		
10–29 min/d	0.68	0.39	1.20	0.50	0.23	1.07	1.03	0.45	2.34
30–119 min/d	1.05	0.66	1.66	0.92	0.49	1.75	1.25	0.68	2.31
120–239 min/d	0.84	0.51	1.39	0.78	0.41	1.50	0.91	0.42	1.95
≥240 min/d	0.75	0.43	1.32	0.61	0.29	1.26	1.02	0.42	2.44
Volume									
VPA									
No activity	1.00			1.00			1.00		
10–74 min/w	1.30	0.30	5.58	1.86	0.40	8.62	N/A		
75–299 min/w	0.40	0.10	1.56	0.14	0.02	1.08	1.34	0.26	6.95
≥300 min/w	0.68 *	0.46	0.99	0.61 *	0.38	0.99	0.84	0.49	1.44
MPA									
No activity	1.00			1.00			1.00		
10–149 min/w	0.34 *	0.18	0.66	0.34 *	0.12	0.92	0.35 *	0.15	0.84
150–299 min/w	0.16 *	0.04	0.69	0.26	0.06	1.17	N/A		
≥300 min/w	0.38 *	0.26	0.55	0.32 *	0.19	0.54	0.47 *	0.29	0.78
LPA									
No activity	1.00			1.00			1.00		
<150 min/w	0.70	0.41	1.21	0.47	0.22	1.01	1.15	0.53	2.50
150–299 min/w	0.54	0.15	1.99	0.63	0.13	3.15	0.37	0.05	2.85
≥300 min/w	0.97	0.64	1.45	0.86	0.50	1.48	1.13	0.63	2.03

Abbreviations: OR, odds ratio; CI, confidence interval; PA, physical activity; VPA, vigorous physical activity; MPA, moderate physical activity; LPA, light physical activity; OR was adjusted for age, sex, educational status, marital status, drinking, smoking, BMI, and case weights; Model 1 was established for the total participants; Model 2 and model 3 were established for men and women, respectively; N/A denoted that no applicable value was observed; *: *p* < 0.05.

## Appendix A

**Table A1:** Associations between PA and the risk of PA (excluding participants with disability).

Variables	Model 4 (*n* = 5748)		
	OR	95% CI	
Frequency			
VPA			
No activity	1.00		
1–2 d/w	1.30	0.65	2.63
3–5 d/w	0.29 *	0.10	0.80
6–7 d/w	0.80	0.49	1.31
MPA			
No activity	1.00		
1–2 d/w	0.35 *	0.15	0.82
3–5 d/w	0.26 *	0.12	0.57
6–7 d/w	0.43 *	0.27	0.66
LPA			
No activity	1.00		
1–2 d/w	0.92	0.33	2.55
3–5 d/w	1.00	0.52	1.92
6–7 d/w	0.94	0.59	1.49
Duration			
VPA			
No activity	1.00		
10–29 min/d	0.88	0.21	3.81
30–119 min/d	0.57	0.20	1.57
120–239 min/d	1.03	0.54	1.94
≥240 min/d	0.66	0.39	1.11
MPA			
No activity	1.00		
10–29 min/d	0.41 *	0.19	0.88
30–119 min/d	0.31 *	0.17	0.55
120–239 min/d	0.42 *	0.23	0.76
≥240 min/d	0.47 *	0.26	0.85
LPA			
No activity	1.00		
10–29 min/d	0.78	0.42	1.44
30–119 min/d	1.07	0.63	1.80
120–239 min/d	0.87	0.49	1.54
≥240 min/d	0.78	0.41	1.48
Volume			
VPA			
No activity	1.00		
10–74 min/w	2.00	0.45	8.87
75–299 min/w	0.57	0.15	2.26
≥300 min/w	0.74	0.47	1.18
MPA			
No activity	1.00		
10–149 min/w	0.38 *	0.18	0.80
150–299 min/w	0.20 *	0.05	0.88
≥300 min/w	0.40 *	0.26	0.62
LPA			
No activity	1.00		
10–149 min/w	0.78	0.42	1.43
150–299 min/w	0.74	0.20	2.79
≥300 min/w	0.98	0.62	1.55

Abbreviations: OR, odds ratio; CI, confidence interval; PA, physical activity; VPA, vigorous physical activity; MPA, moderate physical activity; LPA, light physical activity. OR was adjusted for age, sex, educational status, marital status, drinking, smoking, BMI and case weights. *: *p* < 0.05.

**Table A2:** *E*-values for the point estimates.

Variables	Model 1 (*n* = 6250)			*E*-value	
	OR	95% CI		Point	CI
Frequency					
VPA					
No activity	1.00				
1–2 d/w	0.92	0.46	1.81	-	-
3–5 d/w	0.32 *	0.14	0.75	5.70	2.00
6–7 d/w	0.74	0.49	1.11	-	-
MPA					
No activity	1.00				
1–2 d/w	0.35 *	0.18	0.72	5.16	2.12
3–5 d/w	0.23 *	0.12	0.46	8.16	3.77
6–7 d/w	0.40 *	0.27	0.58	4.44	2.84
LPA					
No activity	1.00				
1–2 d/w	0.74	0.29	1.90	-	-
3–5 d/w	0.89	0.49	1.60	-	-
6–7 d/w	0.92	0.61	1.39	-	-
Duration					
VPA					
No activity	1.00				
10–29 min/d	0.60	0.14	2.55	-	-
30–119 min/d	0.60	0.26	1.37	-	-
120–239 min/d	0.82	0.46	1.45	-	-
≥240 min/d	0.60 *	0.38	0.94	2.72	1.32
MPA					
No activity	1.00				
10–29 min/d	0.31 *	0.14	0.65	5.91	2.45
30–119 min/d	0.32 *	0.20	0.53	5.70	3.18
120–239 min/d	0.40 *	0.24	0.67	4.44	2.35
≥240 min/d	0.40 *	0.23	0.68	4.44	2.30
LPA					
No activity	1.00				
10–29 min/d	0.68	0.39	1.20	-	-
30–119 min/d	1.05	0.66	1.66	-	-
120–239 min/d	0.84	0.51	1.39	-	-
≥240 min/d	0.75	0.43	1.32	-	-
Volume					
VPA					
No activity	1.00				
10–74 min/w	1.30	0.30	5.58	-	-
75–299 min/w	0.40	0.10	1.56	-	-
≥300 min/w	0.68 *	0.46	0.99	2.30	1.11
MPA					
No activity	1.00				
10–149 min/w	0.34 *	0.18	0.66	5.33	2.40
150–299 min/w	0.16 *	0.04	0.69	11.98	2.26
≥300 min/w	0.38 *	0.26	0.55	4.70	3.04
LPA					
No activity	1.00				
10–149 min/w	0.70	0.41	1.21	-	-
150–299 min/w	0.54	0.15	1.99	-	-
≥300 min/w	0.97	0.64	1.45	-	-

Abbreviations: OR, odds ratio; CI, confidence interval; VPA, vigorous physical activity; MPA, moderate physical activity; LPA, light physical activity. OR was adjusted for age, sex, educational status, marital status, drinking, smoking, BMI and case weights. *: *p* < 0.05.

## References

[1] Naghavi M., Abajobir A.A., Abbafati C., Abbas K.M., Abd-Allah F., Abera S.F., Aboyans V., Adetokunboh O., Afshin A., Agrawal A. (2017). Global, regional, and national age-sex specific mortality for 264 causes of death, 1980–2016: A systematic analysis for the Global Burden of Disease Study 2016. Lancet.

[2] Avan A., Digaleh H., Di Napoli M., Stranges S., Behrouz R., Shojaeianbabaei G., Amiri A., Tabrizi R., Mokhber N., Spence J.D. (2019). Socioeconomic status and stroke incidence, prevalence, mortality, and worldwide burden: An ecological analysis from the Global Burden of Disease Study 2017. BMC Med..

[3] Global Burden of Disease Stroke Expert Group (2018). Global, Regional, and Country-Specific Lifetime Risks of Stroke, 1990 and 2016. N. Engl. J. Med..

[4] Wang Y.J., Li Z.X., Gu H.Q., Zhai Y., Jiang Y., Zhao X.Q., Wang Y.L., Yang X., Wang C.J., Meng X. (2020). China Stroke Statistics 2019: A Report From the National Center for Healthcare Quality Management in Neurological Diseases, China National Clinical Research Center for Neurological Diseases, the Chinese Stroke Association, National Center for Chronic and Non-communicable Disease Control and Prevention, Chinese Center for Disease Control and Prevention and Institute for Global Neuroscience and Stroke Collaborations. Stroke Vasc. Neurol..

[5] Wang W., Jiang B., Sun H., Ru X., Sun D., Wang L., Wang L., Jiang Y., Li Y., Wang Y. (2017). Prevalence, Incidence, and Mortality of Stroke in China: Results from a Nationwide Population-Based Survey of 480 687 Adults. Circulation.

[6] O’Donnell M.J., Chin S.L., Rangarajan S., Xavier D., Liu L., Zhang H., Rao-Melacini P., Zhang X., Pais P., Agapay S. (2016). Global and regional effects of potentially modifiable risk factors associated with acute stroke in 32 countries (INTERSTROKE): A case-control study. Lancet.

[7] (2010). WHO Guidelines Approved by the Guidelines Review Committee. Global Recommendations on Physical Activity for Health.

[8] Mora S., Cook N., Buring J.E., Ridker P.M., Lee I.M. (2007). Physical activity and reduced risk of cardiovascular events: Potential mediating mechanisms. Circulation.

[9] Fiuza-Luces C., Santos-Lozano A., Joyner M., Carrera-Bastos P., Picazo O., Zugaza J.L., Izquierdo M., Ruilope L.M., Lucia A. (2018). Exercise benefits in cardiovascular disease: Beyond attenuation of traditional risk factors. Nat. Rev. Cardiol..

[10] Lear S.A., Hu W., Rangarajan S., Gasevic D., Leong D., Iqbal R., Casanova A., Swaminathan S., Anjana R.M., Kumar R. (2017). The effect of physical activity on mortality and cardiovascular disease in 130,000 people from 17 high-income, middle-income, and low-income countries: The PURE study. Lancet.

[11] Kyu H.H., Bachman V.F., Alexander L.T., Mumford J.E., Afshin A., Estep K., Veerman J.L., Delwiche K., Iannarone M.L., Moyer M.L. (2016). Physical activity and risk of breast cancer, colon cancer, diabetes, ischemic heart disease, and ischemic stroke events: Systematic review and dose-response meta-analysis for the Global Burden of Disease Study 2013. BMJ.

[12] Soares-Miranda L., Siscovick D.S., Psaty B.M., Longstreth W.T., Mozaffarian D. (2016). Physical Activity and Risk of Coronary Heart Disease and Stroke in Older Adults: The Cardiovascular Health Study. Circulation.

[13] Kiely D.K., Wolf P.A., Cupples L.A., Beiser A.S., Kannel W.B. (1994). Physical activity and stroke risk: The Framingham Study. Am. J. Epidemiol..

[14] Yu L., Liang Q., Zhou W., Huang X., Hu L., You C., Li J., Wu Y., Li P., Wu Q. (2018). Association between physical activity and stroke in a middle-aged and elderly Chinese population. Medicine (Baltimore).

[15] Zhuang Z., Gao M., Yang R., Li N., Liu Z., Cao W., Huang T. (2020). Association of physical activity, sedentary behaviours and sleep duration with cardiovascular diseases and lipid profiles: A Mendelian randomization analysis. Lipids Health Dis..

[16] Chomistek A.K., Henschel B., Eliassen A.H., Mukamal K.J., Rimm E.B. (2016). Frequency, Type, and Volume of Leisure-Time Physical Activity and Risk of Coronary Heart Disease in Young Women. Circulation.

[17] Jeong H.G., Kim D.Y., Kang D.W., Kim B.J., Kim C.K., Kim Y., Yang W., Park E.S., Lee S.H. (2017). Physical Activity Frequency and the Risk of Stroke: A Nationwide Cohort Study in Korea. J. Am. Heart Assoc..

[18] Zhao Y., Hu Y., Smith J.P., Strauss J., Yang G. (2014). Cohort profile: The China Health and Retirement Longitudinal Study (CHARLS). Int. J. Epidemiol..

[19] VanderWeele T.J., Ding P. (2017). Sensitivity Analysis in Observational Research: Introducing the E-Value. Ann. Intern. Med..

[20] Shepherd A.I., Pulsford R., Poltawski L., Forster A., Taylor R.S., Spencer A., Hollands L., James M., Allison R., Norris M. (2018). Physical activity, sleep, and fatigue in community dwelling Stroke Survivors. Sci. Rep..

[21] Bernhardt J., Dewey H., Thrift A., Donnan G. (2004). Inactive and alone: Physical activity within the first 14 days of acute stroke unit care. Stroke.

[22] Gao F., Zhang L.Q. (2008). Altered contractile properties of the gastrocnemius muscle poststroke. J. Appl. Physiol..

[23] Lieber R.L., Ward S.R. (2013). Cellular mechanisms of tissue fibrosis. 4. Structural and functional consequences of skeletal muscle fibrosis. Am. J. Physiol. Cell Physiol..

[24] Lee S.S., Spear S., Rymer W.Z. (2015). Quantifying changes in material properties of stroke-impaired muscle. Clin. Biomech. (Bristol Avon).

[25] Willey J.Z., Moon Y.P., Paik M.C., Boden-Albala B., Sacco R.L., Elkind M.S. (2009). Physical activity and risk of ischemic stroke in the Northern Manhattan Study. Neurology.

[26] Sattelmair J.R., Kurth T., Buring J.E., Lee I.M. (2010). Physical activity and risk of stroke in women. Stroke.

[27] Shangren Q., Zirui H., Ye D. (2019). Income-Related Inequalities in Chronic Disease Situation Among the Chinese Population Aged Above 45 Years. Inquiry.

[28] Wang Z., Chen Z., Zhang L., Wang X., Hao G., Zhang Z., Shao L., Tian Y., Dong Y., Zheng C. (2018). Status of Hypertension in China: Results From the China Hypertension Survey, 2012-2015. Circulation.

[29] Yang L., Shao J., Bian Y., Wu H., Shi L., Zeng L., Li W., Dong J. (2016). Prevalence of type 2 diabetes mellitus among inland residents in China (2000-2014): A meta-analysis. J. Diabetes Investig..

[30] Borjesson M., Onerup A., Lundqvist S., Dahlof B. (2016). Physical activity and exercise lower blood pressure in individuals with hypertension: Narrative review of 27 RCTs. Br. J. Sports Med..

[31] Smith A.D., Crippa A., Woodcock J., Brage S. (2016). Physical activity and incident type 2 diabetes mellitus: A systematic review and dose-response meta-analysis of prospective cohort studies. Diabetologia.

[32] Zeng Z., Bian Y., Cui Y., Yang D., Wang Y., Yu C. (2020). Physical Activity Dimensions and Its Association with Risk of Diabetes in Middle and Older Aged Chinese People. Int. J. Environ. Res. Public Health.

[33] Aigner A., Grittner U., Rolfs A., Norrving B., Siegerink B., Busch M.A. (2017). Contribution of Established Stroke Risk Factors to the Burden of Stroke in Young Adults. Stroke.

[34] Kubota Y., Iso H., Yamagishi K., Sawada N., Tsugane S., Group J.S. (2017). Daily Total Physical Activity and Incident Stroke: The Japan Public Health Center-Based Prospective Study. Stroke.

[35] Wendel-Vos G.C., Schuit A.J., Feskens E.J., Boshuizen H.C., Verschuren W.M., Saris W.H., Kromhout D. (2004). Physical activity and stroke. A meta-analysis of observational data. Int. J. Epidemiol..

[36] Wang Y., Dai Y., Zheng J., Xie Y., Guo R., Guo X., Sun G., Sun Z., Sun Y., Zheng L. (2019). Sex difference in the incidence of stroke and its corresponding influence factors: Results from a follow-up 8.4 years of rural China hypertensive prospective cohort study. Lipids Health Dis..

[37] Starmer A.J., Frintner M.P., Matos K., Somberg C., Freed G., Byrne B.J. (2019). Gender Discrepancies Related to Pediatrician Work-Life Balance and Household Responsibilities. Pediatrics.

[38] Adjei N.K., Brand T. (2018). Investigating the associations between productive housework activities, sleep hours and self-reported health among elderly men and women in western industrialised countries. BMC Public Health.

[39] Cao X.L., Wang S.B., Zhong B.L., Zhang L., Ungvari G.S., Ng C.H., Li L., Chiu H.F., Lok G.K., Lu J.P. (2017). The prevalence of insomnia in the general population in China: A meta-analysis. PLoS ONE.

[40] Wang Y., Huang W., O’Neil A., Lan Y., Aune D., Wang W., Yu C., Chen X. (2020). Association between sleep duration and mortality risk among adults with type 2 diabetes: A prospective cohort study. Diabetologia.

[41] Dohrn I.M., Welmer A.K., Hagstromer M. (2019). Accelerometry-assessed physical activity and sedentary time and associations with chronic disease and hospital visits - a prospective cohort study with 15 years follow-up. Int. J. Behav. Nutr. Phys. Act..

[42] LaCroix A.Z., Bellettiere J., Rillamas-Sun E., Di C., Evenson K.R., Lewis C.E., Buchner D.M., Stefanick M.L., Lee I.M., Rosenberg D.E. (2019). Association of Light Physical Activity Measured by Accelerometry and Incidence of Coronary Heart Disease and Cardiovascular Disease in Older Women. JAMA Netw. Open.

[43] Hupin D., Raffin J., Barth N., Berger M., Garet M., Stampone K., Celle S., Pichot V., Bongue B., Barthelemy J.C. (2019). Even a Previous Light-Active Physical Activity at Work Still Reduces Late Myocardial Infarction and Stroke in Retired Adults Aged>65 Years by 32%: The PROOF Cohort Study. Front. Public Health.

[44] LaMonte M.J., Buchner D.M., Rillamas-Sun E., Di C., Evenson K.R., Bellettiere J., Lewis C.E., Lee I.M., Tinker L.F., Seguin R. (2018). Accelerometer-Measured Physical Activity and Mortality in Women Aged 63 to 99. J. Am. Geriatr. Soc..

